# Combination of autologous FCS and MPG versus each technique alone in the treatment of stable vitiligo

**DOI:** 10.1007/s00403-024-03045-6

**Published:** 2024-06-08

**Authors:** Mohamed Ibrahim ElGhareeb, Abdullah Hassan Kandeel, Sara Ali Attia

**Affiliations:** Dermatology and Venereology, Faculty of Medicine, Zagazig university, Zagazig, Egypt

**Keywords:** FCS, MPG, Stable Vitiligo

## Abstract

Follicular cell suspension (FCS) transplantation is a novel surgical method for treating resistant stable vitiligo, whereas mini punch grafting is an established effective method for treating stable vitiligo. The combination of FCS and mini punch grafting is a better strategy for the treatment of resistant stable vitiligo. The aim of the study was to evaluate the efficacy of follicular cell suspension, mini punch grafting, and a combination of both techniques in the treatment of stable vitiligo. This prospective comparative study was conducted on 48 patients with stable vitiligo. They were divided into three equal groups, including group A (treated with follicular cell suspension), group B (treated with mini punch grafting), and group C (treated with the combination of both techniques). All patients were followed-up for six months for the assessment of their therapeutic response regarding clinical outcomes. By comparing the data of the three studied groups, we found that the difference in the degree of re-pigmentation after one and three months of treatment was not significant. However, the progress of re-pigmentation was significantly different after six months of treatment among the three studied groups (*P* = 0.027). Specifically, re-pigmentation was significantly better in group C than in groups A and B (*P* = 0.037 and 0.017, respectively), but it was not significantly different between groups A and B.

## Introduction

Vitiligo is an acquired depigmenting of the skin [1]. It results from melanocytes destruction due to biochemical, neural, and autoimmune disturbances [2]. Surgical treatment is an important treatment option for stable vitiligo. Functioning normal melanocytes are transferred from apparently normal skin of vitiligo patient to stable vitiliginous patches through either tissue or cellular grafting. Tissue grafts include mini-punch grafts (MPG), suction blister epidermal grafts (SBEG), split-thickness skin grafts (STSG), and hair follicle grafts. Cellular grafts include autologous follicular and epidermal cell suspension transplantation [3].

Autologous non-cultured outer root sheath (ORS) hair follicle cell suspension, also known as follicular cell suspension (FCS), is a novel surgical therapy for the treatment of vitiligo [4].

Hair follicle melanocytes promote the repigmentation of vitiliginous lesions. Repigmentation in vitiligo can occur in four clinical patterns: follicular, marginal, diffuse, and combined, but follicular is the most common type. In this type of repigmentation, melanocyte stem cells (MelSCs) in the hair follicle is used as a reservoir [5].

MelSCs are present in ORS and can migrate to the epidermis to cause perifollicular repigmentation. These cells later spread in a concentric pattern causing diffuse repigmentation [6].

## Patients and methods

This prospective comparative study was conducted on 48 patients after obtaining written consent from all patients. The protocol of the study was approved by the local institutional review board with reference number: ZU-IRB#6400/13-9-2020. Patients who were at least 18 years old, with stable vitiligo, without the development of new lesions or expansion of the existing lesions over the previous year, and not responding to medical treatment or phototherapy were included in this study. Patients with active vitiligo, a history of Koebnerization, a large portion of the body surface affected (> 50%), pregnancy, keloid tendency, active infection, or bleeding diathesis were excluded from the study. The patients were divided randomly into three equal groups, which included group A : treated with FCS, group B : treated with MPG, and group C: treated with the combination of both techniques.

**Fig. 1:**
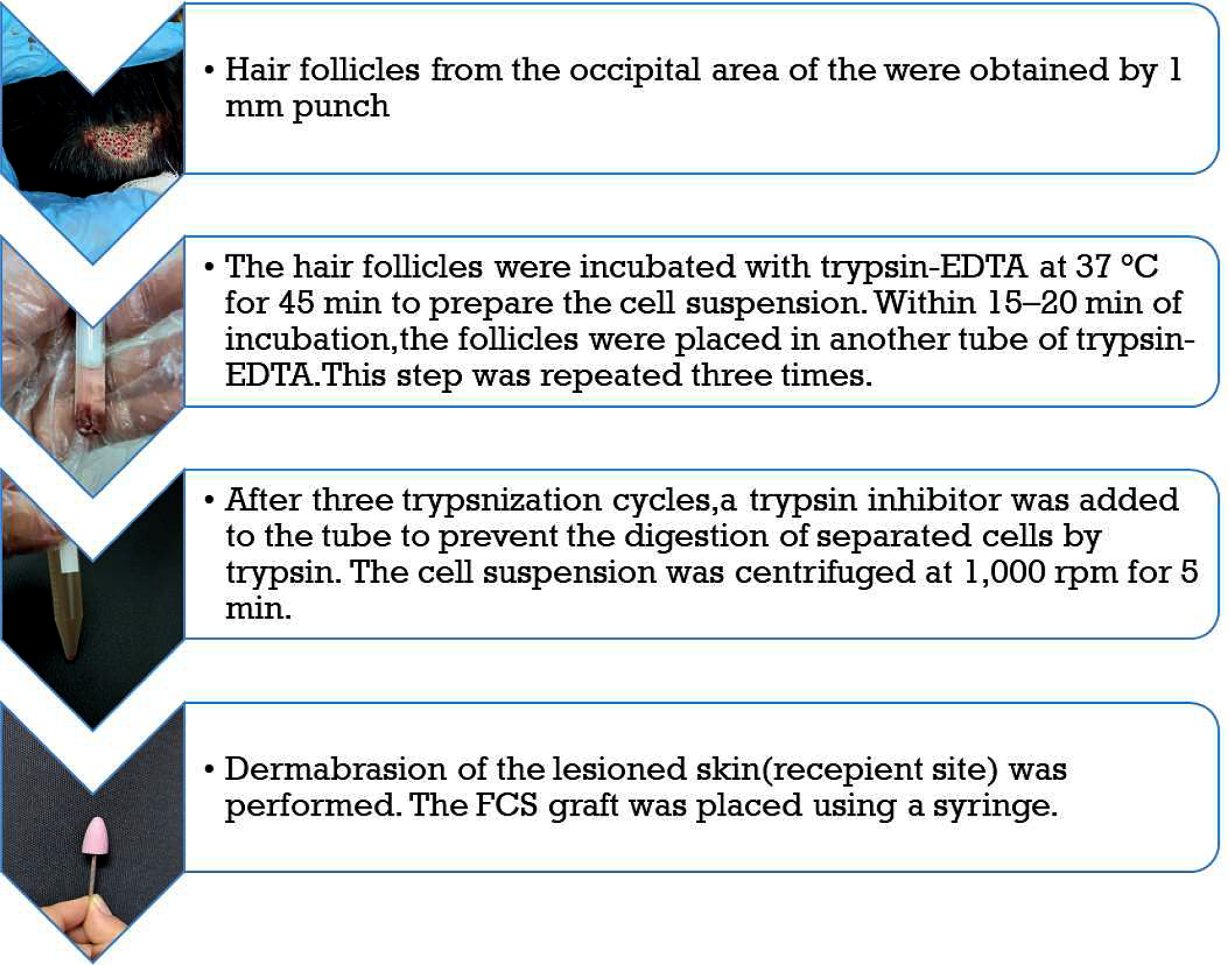
A workflow diagram illustrating steps of FCS preparation

Group A patients were treated by FCS grafting as follow: A few hair shafts from the patient’s occipital scalp were trimmed to 2 mm, then hair follicles were obtained by 1 mm punch. The follicles were incubated with trypsin-EDTA at 37 °C for 45 min to prepare the cell suspension. Within 15–20 min of incubation, the follicles were placed in another tube of trypsin-EDTA.This step was repeated three times. After the three trypsinization cycles, a trypsin inhibitor was added to the tube to prevent the digestion of separated cells by trypsin. The cell suspension was centrifuged at 1,000 rpm for 5 min. The treated vitiliginous patch was dermabraded .The FCS was placed using a syringe. The treated area was covered with sterile vaseline gauze and tape for one week(Fig. 1). Systemic antibiotics (Cephradine)and anti-anaerobes(metronidazole)were given to the patients inorder to prevent secondary bacterial infection.

Group B patients were treated by MPG as follow: Punches of the same size (1.5 mm) were used for the donor and recipient areas. The grafts from the recipient area were removed. The grafts were transplanted directly from the donor to the recipient areas. Dressings were placed over the recipient and donor areas for one week. Systemic antibiotics (Cephradine)and anti-anaerobes(metronidazole)were given to the patients inorder to prevent secondary bacterial infection. Group C patients were treated by combination of both techniques.

Evaluation of treatment response: The patients were examined on the eighth day and at one, three, and six months after the procedure. During each visit, photographs were taken, which were evaluated by two independent dermatologists who were blinded to treatment assignments for assessing the degree of repigmentation. The repigmentation response was graded following the method described by Sahni et al. [7] as follow: poor repigmentation (≤ 25%), fair repigmentation (26–50%), good repigmentation (51–75%), very good repigmentation (76–90%), and excellent repigmentation (> 90%).

## Results

In total, 48 patients participated in the study and were divided into three equal groups. The difference among the groups regarding their baseline characteristics was not significant (Table 1).

**Table 1:** Demographic data and disease characteristics of the studied groups at baseline

		Group A (*n* = 16)	Group B (*n* = 16)	Group C (*n* = 16)	*P* value
*Age * *(years)*	Median	24	29.5	24	0.948
	IQR	20.25–36.25	20–40.25	20–42.25	
*Sex*	Male	6 (37.5%)	11 (68.75%)	7 (43.75%)	0.174
	Female	10 (62.5%)	5 (31.25%)	9 (56.25%)	
*Family history of vitiligo*					
Yes		4 (25%)	3 (18.75%)	3 (18.75%)	0.881
No		12 (75%)	13 (81.25%)	13 (81.25%)	
*Skin type*					
II		1 (6.25%)	0 (0%)	2 (12.5%)	0.572
III		3 (18.75%)	3 (18.75%)	5 (31.25%)	
IV		8 (50%)	10 (62.5%)	8 (50%)	
V		4 (25%)	3 (18.75%)	1 (6.25%)	
*Type of vitiligo*					
Segmental		12 (75%)	14 (87.5%)	14 (87.5%)	0.549
Non segmental		4 (25%)	2 (12.5%)	2 (12.5%)	
*Duration (years)*					
Median		7	8	7	0.688
IQR		5–10	5.25–11.75	5.25–10	
*Stability (years)*					
Median		2.5	3	3	0.172
IQR		2–3	2.25–5	2.25–3.75	

**Fig. 2:**
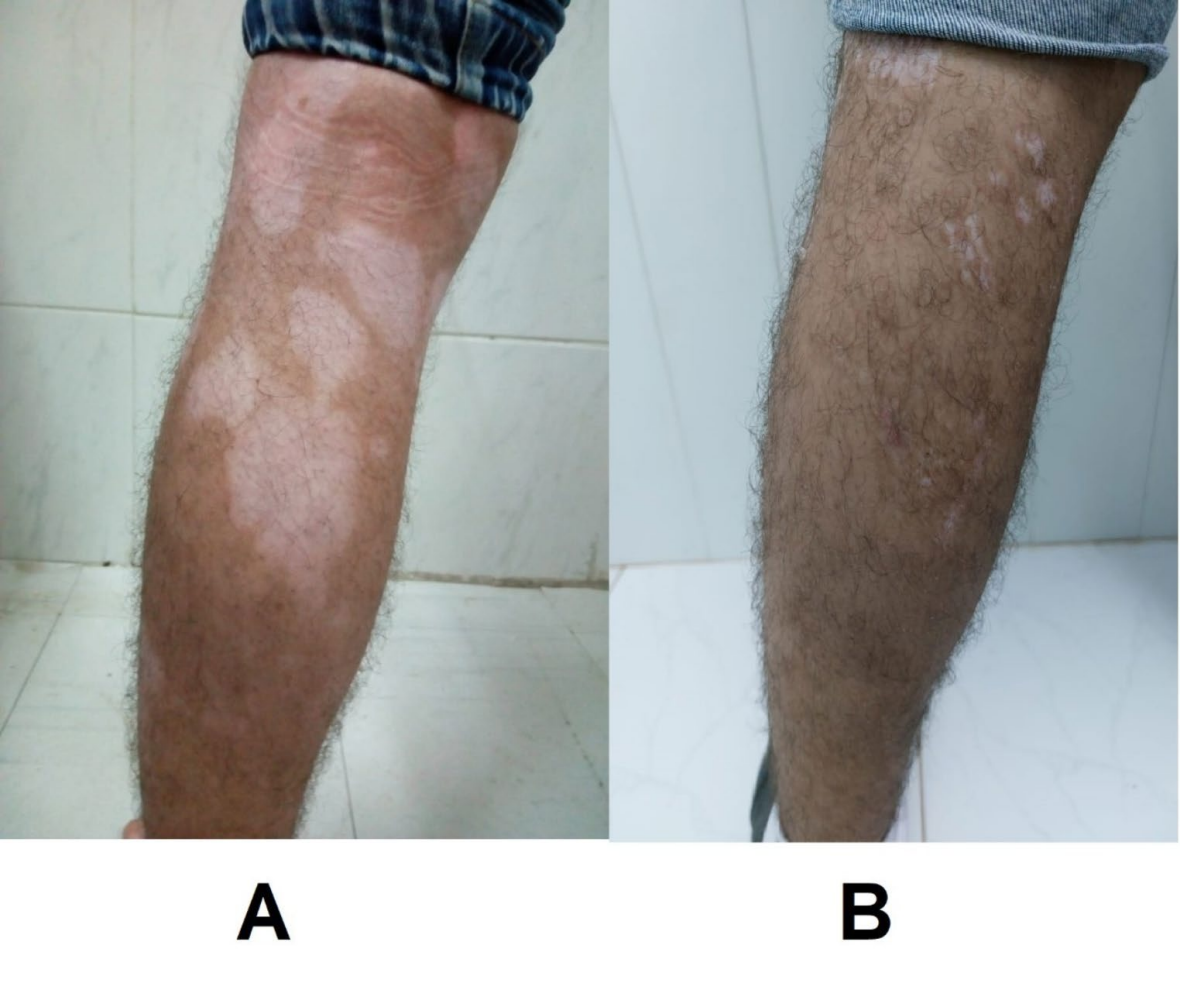
FCS at back of leg (**A**): before treatment, (**B**): at 6 months after treatment

**Fig. 3:**
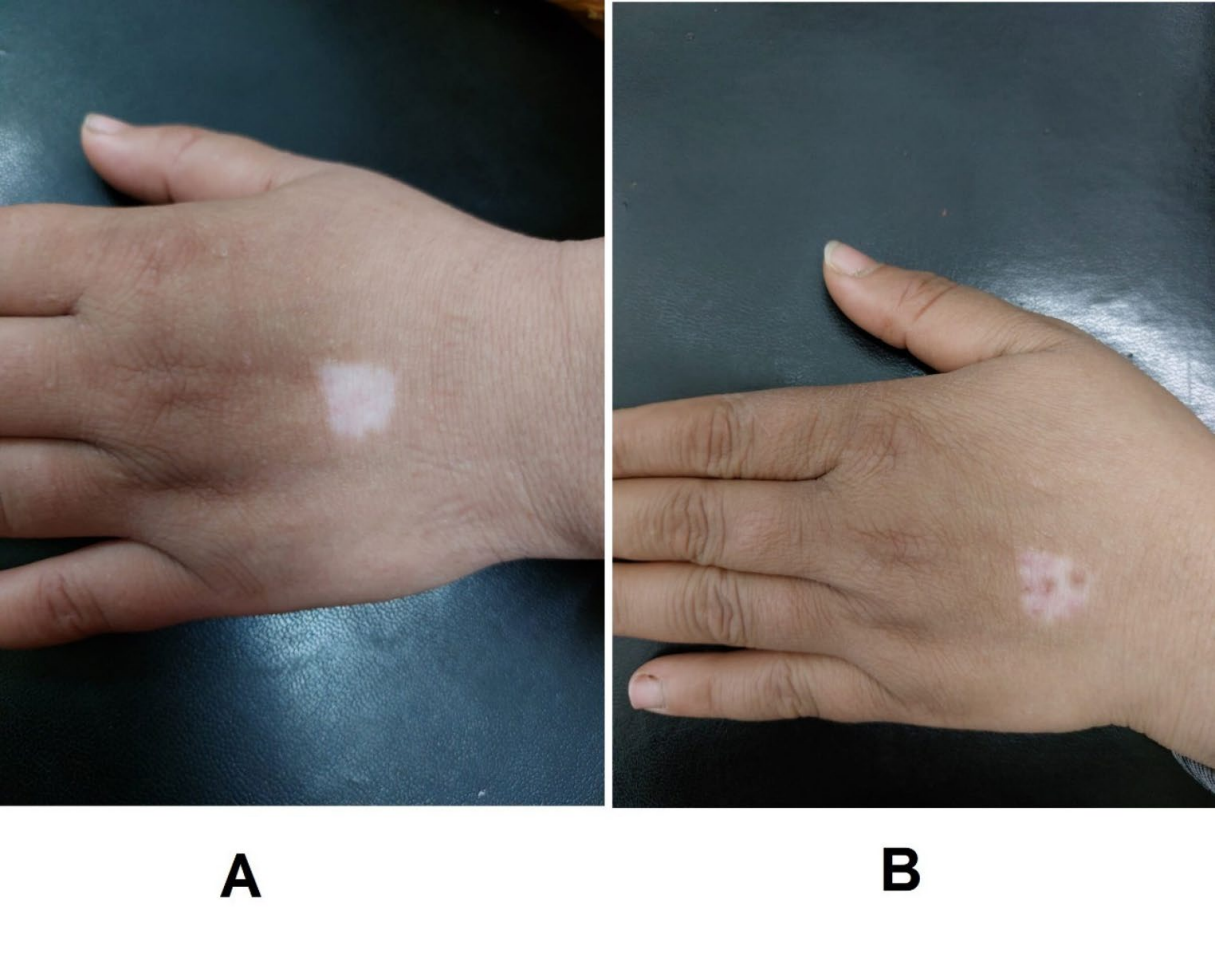
MPG at dorsum of hand (**A**): before treatment, (**B**): at 6 months after treatment

**Fig. 4:**
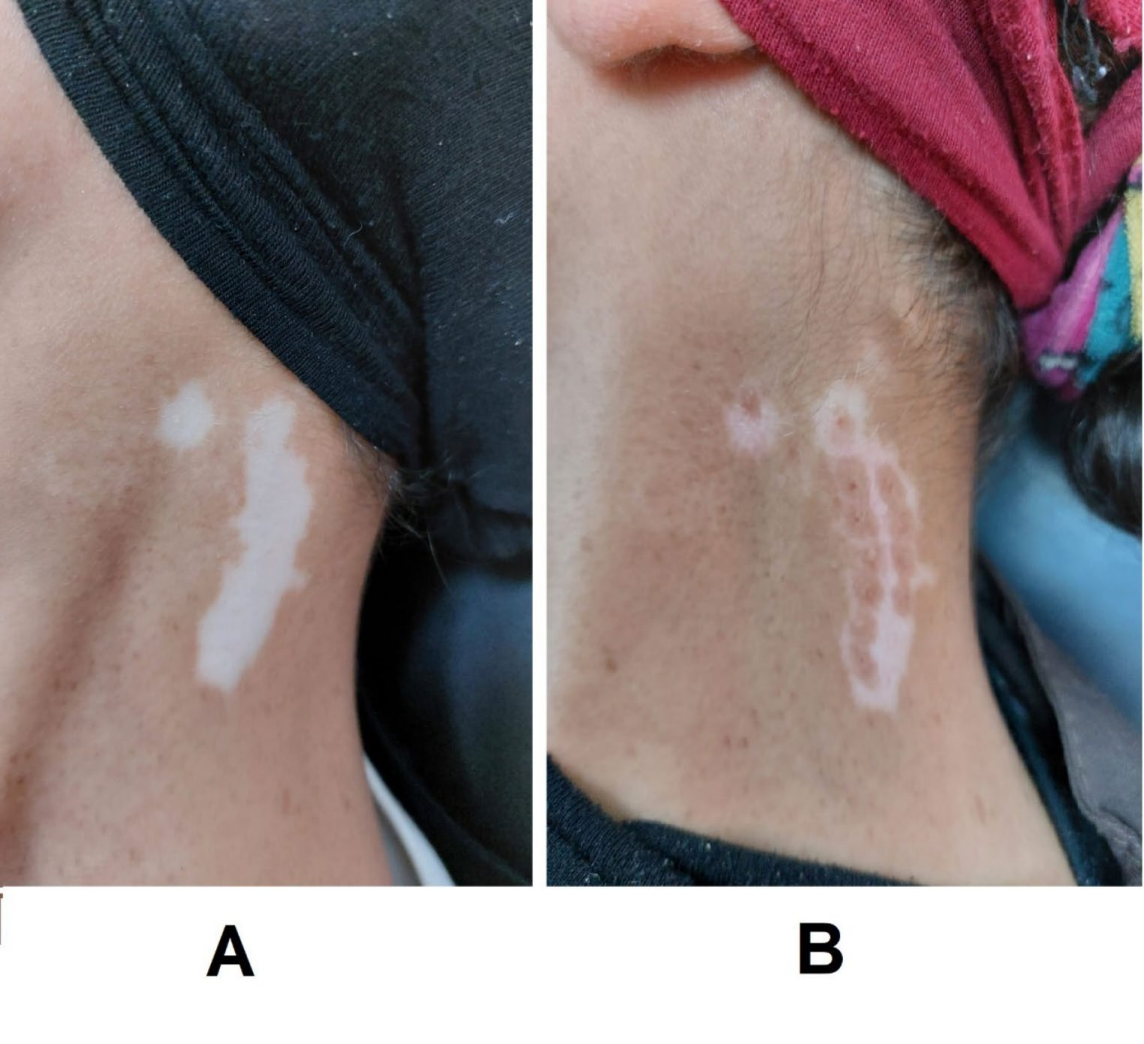
Combination of FCS and MPG at side of neck (**A**) :before treatment, (**B**) :at 6 months after treatment

By comparing the three studied groups, we found that the difference in the degree of re-pigmentation was not significant after one and three months of treatment. However, the progress of repigmentation was significantly different after six months of treatment among the three studied groups (*P* = 0.027). Re-pigmentation was significantly better in group C compared to that in groups A and B (*P* = 0.037 and 0.017, respectively), but it was not significantly different between groups A and B (Table 2), (Figs. 2, 3 and 4).

**Table 2:** Comparison between the three studied groups regarding re-pigmentation over follow-up periods

	Group A (*n* = 16)	Group B (*n* = 16)	Group C (*n* = 16)	*p* value
*1 month*				
Poor	3 (18.75%)	5 (31.25%)	2 (12.5%)	0.64
Fair	5 (31.25%)	6 (37.5%)	3 (18.75%)	
Good	3 (18.75%)	3 (18.75%)	4 (25%)	
Very good	3 (18.75%)	2 (12.5%)	4 (25%)	
Excellent	2 (12.5%)	0 (0%)	3 (18.75%)	
*3 months*				
Poor	1 (6.25%)	4 (25%)	1 (6.25%)	0.38
Fair	3 (18.75%)	3 (18.75%)	2 (12.5%)	
Good	4 (25%)	5 (31.25%)	3 (18.75%)	
Very good	4 (25%)	4 (25%)	5 (31.25%)	
Excellent	4 (25%)	0 (0%)	5 (31.25%)	
*6 months*				
Poor	1 (6.25%)	2 (12.5%)	0 (0%)	0.027
Fair	2 (12.5%)	1 (6.25%)	0 (0%)	
Good	5 (31.25%)	6 (37.5%)	1 (6.25%)	
Very good	1 (6.25%)	6 (37.5%)	7 (43.75%)	
Excellent	7 (43.75%)	1 (6.25%)	8 (50%)	

p1 (Comparison between group A and group B) = 0.066

p2(Comparison between group A and group C) = 0.037

p3 (Comparison between group B and group C) **=** 0.017

Regarding the adverse effects of these techniques, the difference among the three groups was not significant for the incidence of the Halo phenomenon at the recipient site; only one patient in group A and four patients in group B experienced it. No patient in any group suffered from infection or scars at the donor site (Table 3).

**Table 3:** Adverse effects of the three used treatments

	Group A (*n* = 16)	Group B (*n* = 16)	Group C (*n* = 16)	*P* value
At recipient site Halo phenomenon	1 (6.25%)	4 (25%)	0 (0%)	0.055
Infection	0 (0%)	0 (0%)	0 (0%)	---
At donor site Scar	0 (0%)	0 (0%)	0 (0%)	---

## Discussion

A hair follicle is an important reservoir of melanocytes as it has many MelSCs.Those precursor cells are present in ORS and mid-follicle and the matrix of the hair bulb [8].This reservoir of melanocytes is important for the treatment of vitiligo [9].

Significant repigmentation in most of our patients indicated that the treatment method proposed by us was effective. The method can be used for treating large depigmented areas. In the follicular melanin unit, there is one melanocyte for every five keratinocytes in the hair bulb [10],which is considerably higher than that in the epidermal melanin unit, which has one melanocyte for every 36 keratinocytes. Compared to the conventional epidermal cell suspension technique [11],our technique was simpler, as it did not need the separation of the epidermis from the dermis or the manual fragmentation of epidermal samples into small pieces. ORS cells are easily separated when incubated with trypsin. The small scars of follicular unit extraction (FUE) on the scalp were invisible and insignificant. FUE involves the removal of a lesser tissue volume with shorter healing time than that in an epidermal shave biopsy.The cell suspensions used in this study were autologous and required minimal in vitro manipulation. This procedure can serve as a simple, novel, less invasive technique with a good yield of melanocytes, and MelSCs. The limitations of this study were a small sample size and a short follow-up period.

MPG is the easiest, fastest method of vitiligo surgery. In this study, we used punches (1.5 mm) in both donor and recipient areas to decrease the development of a cobblestone appearance.

We used a technique in which FCS and MPG were combined for the treatment of stable vitiligo. This technique was more effective in the repigmentation of vitiliginous patches.Up to our knowledge, this was the first study in which the combination of FCS and MPG was used in the treatment of stable vitiligo.

## Conclusion

The combination of FCS and MPG is a better and more effective for the treatment of stable vitiligo than each technique alone.

## Data Availability

No datasets were generated or analysed during the current study.
